# Supplementary material for: Predictability and epidemic pathways in global outbreaks of infectious diseases: the SARS case study
Source: BMC Med. 2007 Nov 21;5:34. doi: 10.1186/1741-7015-5-34 (PMC2213648; doi:10.1186/1741-7015-5-34)
Supplement: Additional file 1 — Additional figures and materials. The Additional file 1 contains a detailed description of the infection dynamics considered in the model, together with the results of the sensitivity analysis performed on the values of the risk threshold and of the intervention delay. The comparison with the results presented in the manuscript is presented and discussed. [file 1741-7015-5-34-S1.pdf]

Predictability and epidemic pathways in global outbreaks of infectious diseases: the SARS case study.

Additional Material

Vittoria Colizza[1,*], Alain Barrat[1,2], Marc Barthélemy[3],
Alessandro Vespignani[4,5]

1) Complex Networks Lagrange Laboratory (CNLL), Institute for Scientific Interchange (ISI) Foundation, Turin, Italy
2) Unité Mixte de Recherche du CNRS UMR 8627, Bâtiment 210, Univ Paris-Sud, F-91405 Orsay, France
3) CEA-DIF Centre d'Etudes de Bruyères-Le-Châtel, BP12, F- 91680, France
4) School of Informatics and Center for Biocomplexity, Indiana University, Bloomington, IN 47401, USA
5) Institute for Scientific Interchange (ISI) Foundation, Turin, Italy

[*]Corresponding author: Vittoria Colizza, email : vcolizza@isi.it

Discrete stochastic infection dynamics. Here we report the details of the stochastic evolution of the infection dynamics within each city, when the SARS compartmentalization of Figure 1 of the main text is considered. As described in the main text for the SIR model, the change in time of the populations in each compartment is described through binomial and multinomial processes. The SARS transmission model proceeds as follows. Susceptible individuals (S) exposed to SARS enter the latent class. Latents (L) represent infected who are not yet contagious and are assumed to be asymptomatic. After an average latency period of ε^{-1}, they become infectious (I). Individuals are classified as infectious during an average time equal to μ^{-1} from the onset of

clinical symptoms to the admission to the hospital where they eventually die (H_D) or recover (H_R). Patients admitted to the hospital are not allowed to travel, and are considered able to transmit the infection, their infectiousness β reduced by a factor r_β. The average periods spent in the hospital from admission to death or recovery are equal to μ_D^{-1} and μ_R^{-1}, respectively.

As an example of the modeled discrete evolution, here we provide the evolution for the latent compartment. According to the above compartmentalization, the generation of new latents, due to susceptible individuals S coming into contact with infected I and patients H_R and H_D, is described by a multinomial process, whose parameters are given by the probabilities associated to the single processes considered and the population of the given compartment. After the latency period, individuals undergo a transition to infectious (i.e. $L \rightarrow I$ with rate ε), process described through a binomial distribution with a probability given by the rate of transition and number of trials given by the number of latents in the compartment at time t. The number of latents at time t is then given by:

$$L(t+\Delta t) = L(t) + \Delta_{S\rightarrow L} - \Delta_{L\rightarrow I}\,,$$

where $\Delta_{S\rightarrow L}$ and $\Delta_{L\rightarrow I}$ are two integer stochastic variables obtained, respectively: (i) from a multinomial distribution characterized by the probabilities $\left(\frac{\beta I(t)}{N}\Delta t, \frac{r_\beta \beta H_R(t)}{N}\Delta t, \frac{r_\beta \beta H_R(t)}{N}\Delta t, 1 - \frac{\beta\left(I + r_\beta (H_R + H_D)\right)}{N}\Delta t\right)$ out of the total pool of susceptible at time t, $S(t)$; (ii) from a binomial distribution with probability $\varepsilon\Delta t$ and number of trials equal to the number of latents at time t, $L(t)$.

Risk threshold: sensitivity analysis. The main text provides the risk assessment analysis in terms of a risk threshold set to 20%. Here we provide the results obtained by changing the value of the threshold to a lower (10%) and higher (30%) value. Values lower than 10% or higher than 30% would not provide meaningful information since not statistically significant and unrealistic. Figure 1 shows the comparison between empirical data and forecasted results, as obtained with risk thresholds of 10% and 30%.

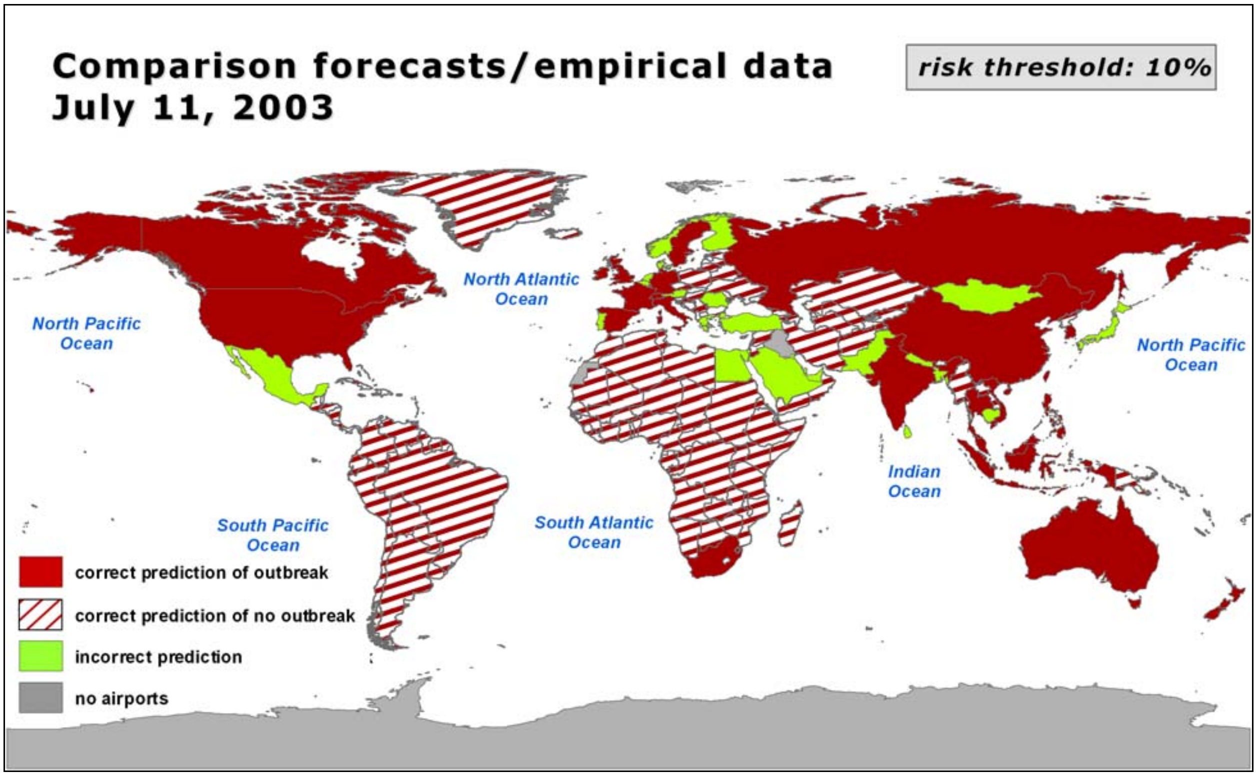

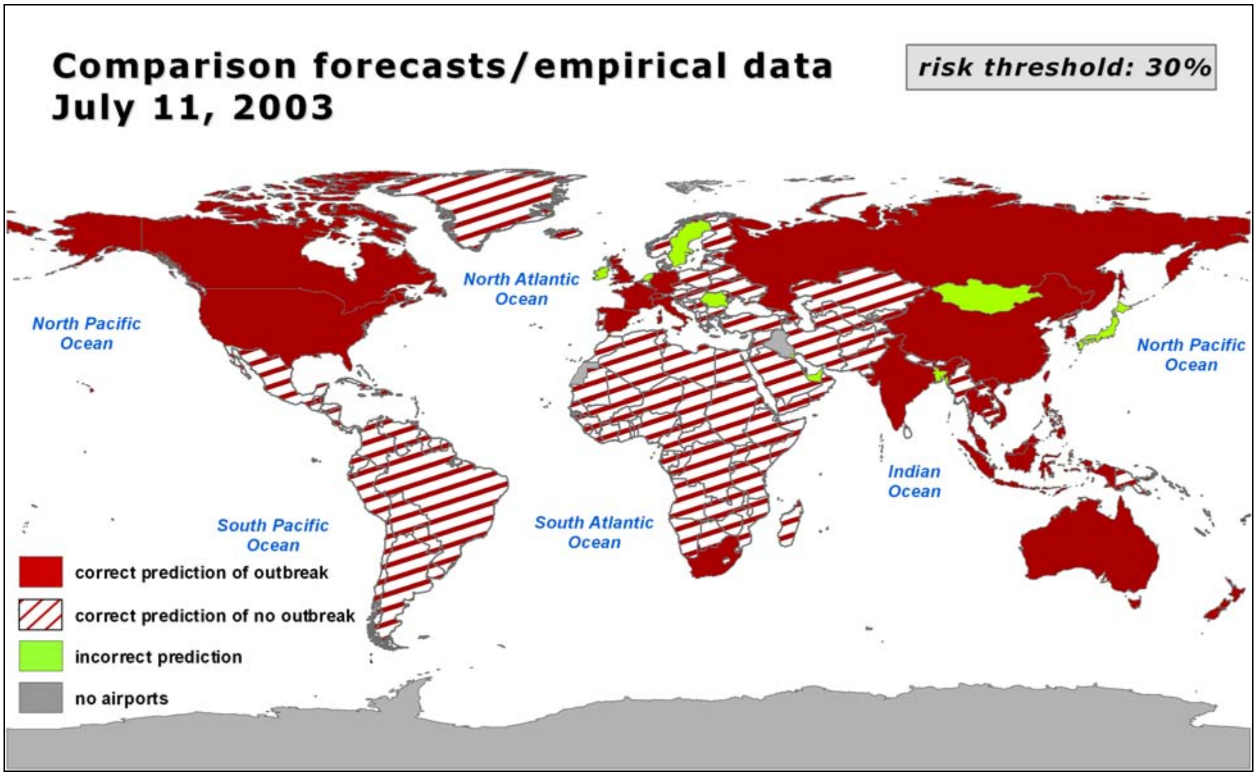

Figure 1. Map representation of the comparison between WHO reported cases and numerical results, with two different values of the risk threshold – 10% and 30%.

The good agreement shown in the main text for a risk threshold of 20% is preserved also here, where countries are defined at risk if their outbreak probability is larger than 10% or 30%. Differences are obviously observed, with an error changing from 12% (26 incorrect predictions out of 220 countries if the risk threshold is set to 10%) to 4% (9 incorrect predictions if the risk threshold is set to 30%), as compared to the 7% error made if we define a country as at risk if its outbreak likelihood is larger than 20%. Overall, variations in the risk threshold do not dramatically change the results of the analysis presented in the main text.

Intervention delay: sensitivity analysis. The results presented in the main text assume a delay of one week from the detection of the first case in the implementation of the intervention strategies aimed at reducing the virus transmissibility. Here we report the results obtained from simulations in which we assume no delay and 2 weeks delay in the intervention. Figure 2 shows the quantitative predictions obtained in the two cases. The no delay case is relevant from the modeling perspective, having no additional parameter to consider, and can be used as a benchmark to test different delays in the implementation of the intervention strategies at the local level. The trend observed by comparing the no delay case with the 1- and 2-weeks delay clearly shows that the model predictions considerably improve for the countries which experienced a large outbreak (Taiwan, Canada, Singapore, Vietnam), while leaving the outbreak magnitude for the other infected countries almost unchanged. A more sophisticated approach could consider intervention delays specific to each country in order to simulate different response times and measures related to local health care systems and policies. The minimal assumption of uniform intervention delay is however able to produce remarkably good results and useful insights in the understanding of the propagation process.

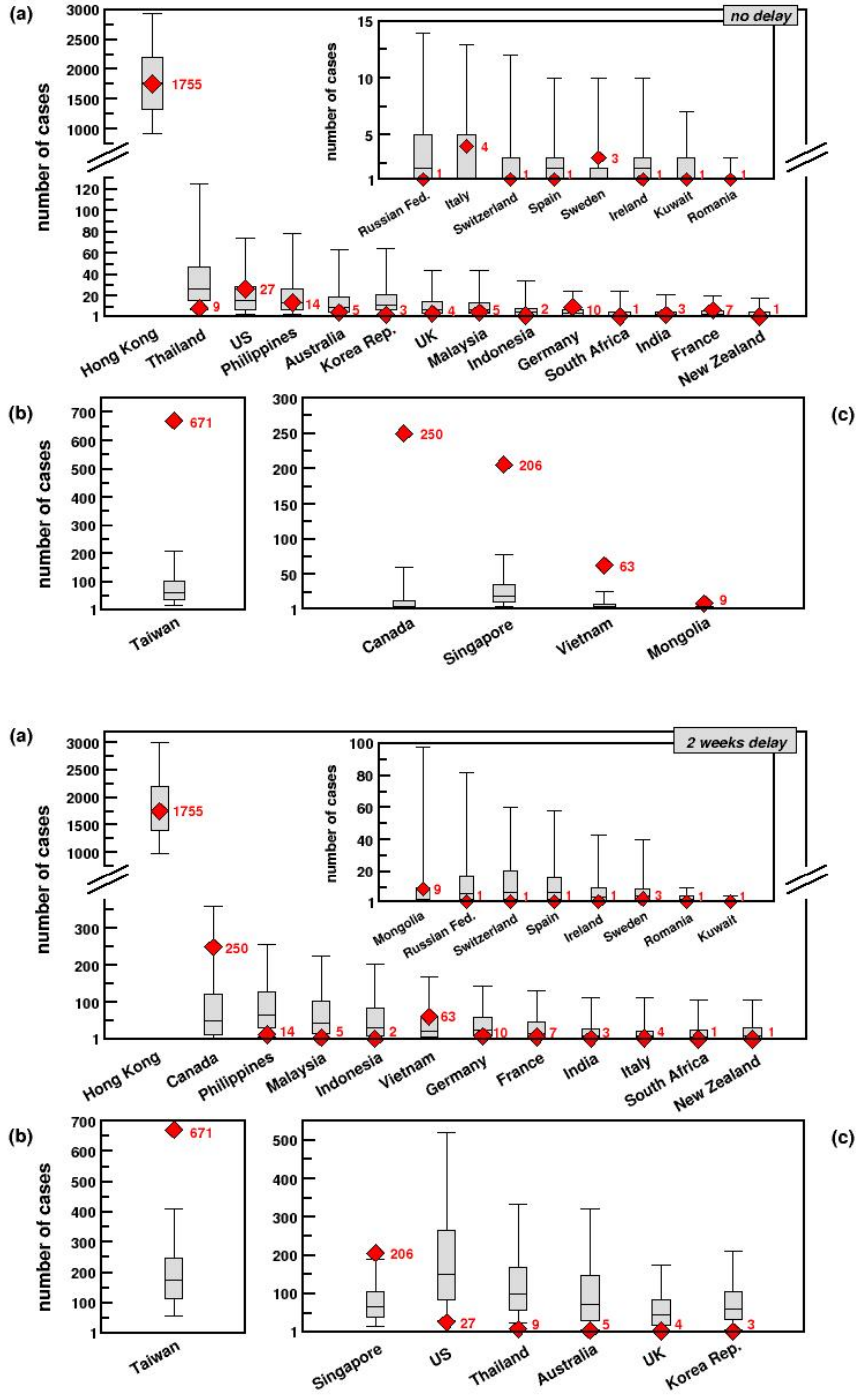

Figure 2. Effect of intervention delay on the outbreak magnitude: no delay (top) and 2 weeks delay (bottom).
